# Lidocaine-Induced Systemic Toxicity: A Case Report and Review of Literature

**DOI:** 10.7759/cureus.1275

**Published:** 2017-05-25

**Authors:** Badar Hasan, Talal Asif, Maryam Hasan

**Affiliations:** 1 Department of Internal Medicine, University of Missouri Kansas City (UMKC); 2 Cardiology, John H. Stroger Hospital of Cook County, Chicago, USA; 3 Internal Medicine, NYU Langone Medical Center, New York

**Keywords:** lidocaine-induced systemic toxicity, local anesthetic toxicity, intravenous lipid emulsion

## Abstract

For the past 50 years, local anesthetics such as lidocaine have been commonly used in various clinical settings. Its use is not just limited to anesthesia and surgery but is also frequently utilized in internal medicine and in primary care setting for bedside procedures. Despite its widespread use, most physicians are not familiar with the life-threatening manifestations of lidocaine toxicity and its treatment. Our case demonstrates a successful resuscitation after cardiac arrest in a healthy 33-year-old female with systemic lidocaine toxicity after she received lidocaine as a local anesthetic. Our goal is to educate general internists and primary care physicians of the possible hazards of lidocaine use. We also aim to create mindfulness of the symptoms of lidocaine toxicity and the use of intravenous lipid emulsion as an antidote.

## Introduction

Local anesthetics are widely used in everyday practice [1]. Their application ranges from use in outpatient medicine clinics to emergency departments and operation theaters [1]. Systemic toxicity from local anesthetics is rarely seen but can be potentially lethal by causing seizures, arrhythmias and cardiovascular collapse [1]. The site of administration and the dose of the local anesthetic delivered are independent risk factors for systemic toxicity [1]. We present a rare case of lidocaine-induced systemic toxicity in a healthy 33-year-old female who underwent an elective procedure as an outpatient. Our aim is to raise awareness among general internists to identify the warning signs of local anesthetic toxicity. We also intend to create an understanding of the pathophysiology behind its various clinical manifestations and the use of intravenous lipid infusion as the treatment of choice to reverse the symptoms.

## Case presentation

A 33-year-old female patient with no significant past medical history presented originally for an elective nasal septoplasty at an outpatient surgical center for a deviated nasal septum. On pre-procedure assessment, her blood pressure was 112/70 mmHg, heart rate 82 beats per minute (bpm), respiratory rate 18 per minute and temperature 98°F. The patient denied having any allergies, alcohol, tobacco or illicit drug use. On cardiovascular examination, she had regular rate and rhythm with no murmurs. Her respiratory, abdominal and neurological exam was also unremarkable.

After preliminary assessment, she was intubated with propofol and succinylcholine. The patient was then given 60 milliliters (ml) of 2% lidocaine with 1% epinephrine subcutaneously in the nasal mucosa. Shortly after receiving the lidocaine, the patient experienced bradycardia followed by a pulseless electrical activity (PEA). Cardiopulmonary resuscitation (CPR) was initiated with the administration of one milligram (mg) of epinephrine a total of three times as per advanced cardiac life support (ACLS) algorithm. Twenty minutes into chest compressions, local anesthetic systemic toxicity was suspected by anesthesia, and the patient was given a 100 ml bolus of 20% intravenous lipid emulsion (intralipids) with restoration of normal sinus rhythm within three minutes of injection. The patient was then placed on the intralipid and norepinephrine infusion and was subsequently transferred to our cardiac intensive care unit (CICU).

**Figure 1:**
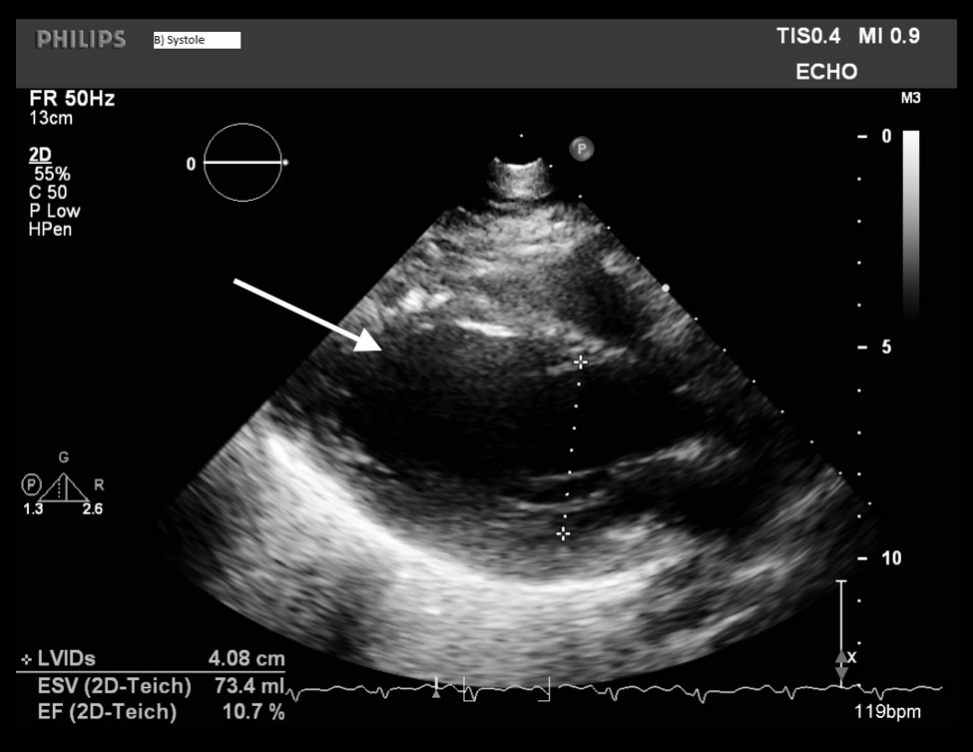
Initial TTE obtained at the time of admission showing an estimated ejection fraction of 10-15%. The arrow shows the dilated left ventricular chamber. TTE - Transthoracic echocardiogram

The patient was hypotensive upon arrival with a blood pressure of 60/40 mmHg and a heart rate of 115 bpm. The initial laboratory evaluation showed a normal complete blood count, comprehensive metabolic panel and troponin T level. The electrocardiogram revealed sinus tachycardia. A chest x-ray was obtained that showed evidence of bilateral pulmonary edema. A transthoracic echocardiogram (TTE) showed a severely reduced left ventricular systolic function with estimated ejection fraction between 10-15% (Figure 1).

No valvular abnormalities were noted.

**Figure 2:**
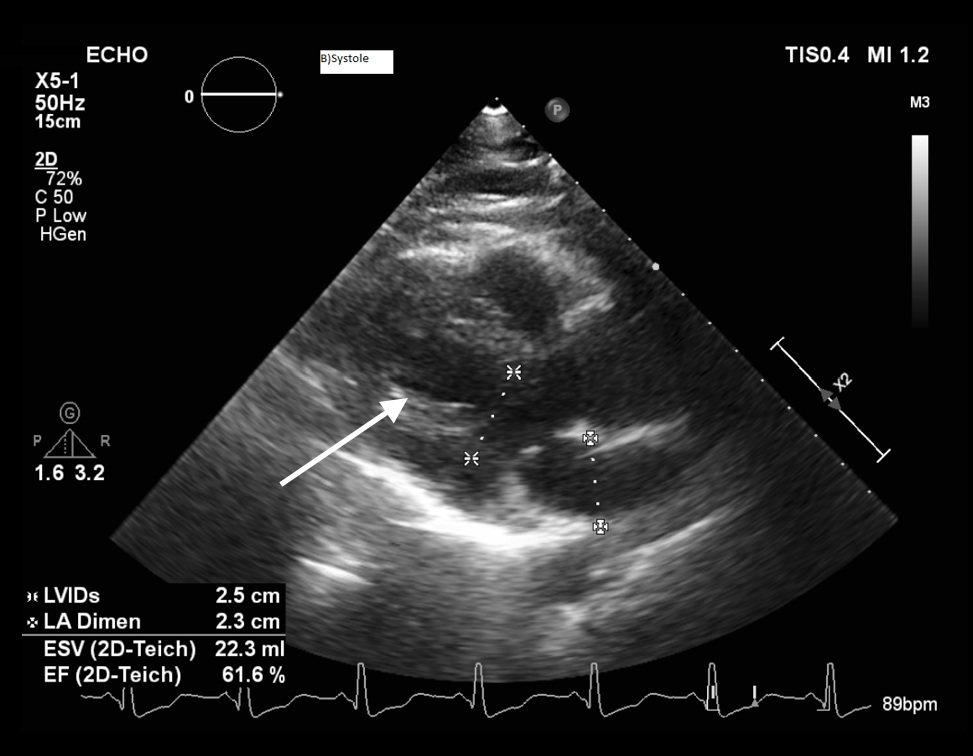
TTE obtained on day 2 of admission showing an improved ejection fraction of 55%. The arrow points to improved left ventricular contraction. TTE - Transthoracic echocardiogram

The patient was started on furosemide drip. She was titrated off norepinephrine and started on dobutamine for inotropic support. The case was also discussed with the poison control center and we were recommended to continue with supportive management and intralipid therapy. Next day, a repeat TTE showed improved an ejection fraction of 55% (Figure 2).

The patient was subsequently weaned off the dobutamine drip and the lipid infusion. She was successfully extubated at the same time as well. A cardiac magnetic resonance imaging looking for infiltrative or inflammatory cardiomyopathy was obtained and was negative. Two days later the patient was discharged and was faring well on follow-up.

## Discussion

Lidocaine is a class 1 B antiarrhythmic agent, which is mainly used for the treatment of ventricular arrhythmias. Since its advent in 1948, it has also become the most commonly used local anesthetic in the outpatient setting. According to a report published in 2013 by the US Poison Control Center, there were 1454 reports of lidocaine exposure with five cases ending up as a fatality [2]. Miscalculation of the dose, injection of the drug into a blood vessel or repeated administration of therapeutic doses are the major causes of systemic toxicity [2-3].

Lidocaine works by binding voltage-gated sodium channels thus inhibiting the propagation of action potential. The main target organs are the central nervous system (CNS) and the cardiovascular system (CVS). Since the CNS is more sensitive to electrophysiological changes than the CVS, neurological symptoms such as dizziness, tinnitus and peri-oral numbness usually precede cardiovascular manifestations. Aburwai, et al. demonstrated lidocaine dose dependent cardiotoxicity in murine cardiomyocytes by inhibiting myocardial cellular respiration. They explained the clinical manifestations of sinus slowing, prolonging of QRS interval, hypotension, shock, and dysrhythmias [4-5]. Separation of sarcomeres, focal myocardial and perivascular fibrosis are the key histological manifestations that have been described in one autopsy report of a suicide case from oral lidocaine ingestion [6].

The diagnosis of lidocaine toxicity is usually clinical as serum levels are not readily available and they do not guide or change treatment. Therapeutic concentrations of lidocaine can be up to 5.5 milligrams per liter (mg/L), whereas a plasma level of 8-12 mg/L and above is associated with CNS and cardiotoxicity [7].

The most critical aspect of local anesthetic is appropriate dosing. The recommended maximum dose for subcutaneous infiltration of lidocaine without epinephrine is 4.5 milligrams per kilogram (mg/kg) and for lidocaine with epinephrine is 7 mg/kg [8]. Asystole is usually seen in patients who receive 800-1000 milligrams (mg) of intravenous lidocaine [1]. Our patient’s weight was 65 kg and she received 60 mL of 2% solution. This totals 1200 mg which is four times the amount of recommended dose of 292 mg in her case.

Once a patient develops cardiac arrest the only therapy that prevents mortality is lipid infusion. It was first demonstrated by Weinberg, et al. in rats during resuscitation after a lethal dose of bupivacaine [9]. American Society of Regional Anesthesia and Pain Medicine recommends an initial bolus of 1.5 mL/kg of 20% intravenous lipids (intralipids) followed by a continuous infusion of 0.25 mL/kg/minute. If cardiac stability is not restored, then the infusion rate can be doubled to 0.5 mL/kg/min, and it is recommended to continue the infusion until cardiac stability is restored for at least 10 minutes. Various mechanisms have been postulated on how lipid infusion works, but the most widely accepted is the “Sink” theory by which lipids create a sink that extracts local anesthetics from plasma making them unavailable for myocardial tissue [5]. Repeated aspiration before each injection is a useful strategy to prevent toxicity [3].

Our patient had no previous cardiac history. She was injected with four times the recommended dose of lidocaine at the anterior aspect of the nasal septum (with a rich blood supply) leading to cardiac arrest. The rapid lipid infusion led to the return of spontaneous circulation and an improvement in the ejection fraction from 15 to 55% over 24 hours, hence preventing morbidity and mortality.

## Conclusions

Systemic toxicity of lidocaine can be life-threatening. The rapid identification of clinical symptoms is key to prevent mortality. Our aim is to ensure that general internists and primary care physicians are able to diagnose and treat lidocaine toxicity. Once a patient develops cardiac arrest, following the general ACLS protocol would not result in successful resuscitation, and administration of intravenous lipid infusion is key to prevent mortality.
