# Correction: Lidocaine-Induced Systemic Toxicity: A Case Report and Review of Literature

**DOI:** 10.7759/cureus.c24

**Published:** 2019-08-23

**Authors:** Badar Hasan, Talal Asif, Maryam Hasan

**Affiliations:** 1 Department of Internal Medicine, University of Missouri Kansas City (UMKC); 2 Cardiology, John H. Stroger Hospital of Cook County, Chicago, USA; 3 Internal Medicine, NYU Langone Medical Center, New York

The discussion section originally contained the following statement: 

"American Society of Regional Anesthesia and Pain Medicine recommends an initial bolus of 1.5 **mg**/kg of 20% intravenous lipids (intralipids) followed by a continuous infusion of 0.25 mL/kg/minute. "

The unit of measurement (in bold above) was incorrectly labeled as "mg" when it should be "mL" - the text of the article has been updated to reflect this correction.

